# SWI/SNF Enzymes Promote SOX10- Mediated Activation of Myelin Gene Expression

**DOI:** 10.1371/journal.pone.0069037

**Published:** 2013-07-16

**Authors:** Himangi G. Marathe, Gaurav Mehta, Xiaolu Zhang, Ila Datar, Aanchal Mehrotra, Kam C. Yeung, Ivana L. de la Serna

**Affiliations:** University of Toledo College of Medicine and Life Sciences, Department of Biochemistry and Cancer Biology, Toledo, Ohio, United States of America; Michigan State University, United States of America

## Abstract

SOX10 is a Sry-related high mobility (HMG)-box transcriptional regulator that promotes differentiation of neural crest precursors into Schwann cells, oligodendrocytes, and melanocytes. Myelin, formed by Schwann cells in the peripheral nervous system, is essential for propagation of nerve impulses. SWI/SNF complexes are ATP dependent chromatin remodeling enzymes that are critical for cellular differentiation. It was recently demonstrated that the BRG1 subunit of SWI/SNF complexes activates SOX10 expression and also interacts with SOX10 to activate expression of OCT6 and KROX20, two transcriptional regulators of Schwann cell differentiation. To determine the requirement for SWI/SNF enzymes in the regulation of genes that encode components of myelin, which are downstream of these transcriptional regulators, we introduced SOX10 into fibroblasts that inducibly express dominant negative versions of the SWI/SNF ATPases, BRM or BRG1. Dominant negative BRM and BRG1 have mutations in the ATP binding site and inhibit gene activation events that require SWI/SNF function. Ectopic expression of SOX10 in cells derived from NIH 3T3 fibroblasts led to the activation of the endogenous Schwann cell specific gene, myelin protein zero (MPZ) and the gene that encodes myelin basic protein (MBP). Thus, SOX10 reprogrammed these cells into myelin gene expressing cells. Ectopic expression of KROX20 was not sufficient for activation of these myelin genes. However, KROX20 together with SOX10 synergistically activated MPZ and MBP expression. Dominant negative BRM and BRG1 abrogated SOX10 mediated activation of MPZ and MBP and synergistic activation of these genes by SOX10 and KROX20. SOX10 was required to recruit BRG1 to the MPZ locus. Similarly, in immortalized Schwann cells, BRG1 recruitment to SOX10 binding sites at the MPZ locus was dependent on SOX10 and expression of dominant negative BRG1 inhibited expression of MPZ and MBP in these cells. Thus, SWI/SNF enzymes cooperate with SOX10 to directly activate genes that encode components of peripheral myelin.

## Introduction

Glial cells insulate axons by forming a lipid rich structure called the myelin sheath [1]. Two types of myelinating cells, oligodendrocytes in the central nervous system (CNS) and Schwann cells in the peripheral nervous system (PNS) are essential for nervous system development and for proper conduction of nerve impulses. De-myelinating diseases, such as multiple sclerosis of the CNS [2], and neuropathies such as Charcot–Marie–Tooth Disease of the PNS cause severe sensory and motor defects [3]. Inherited neuropathies of the PNS are characterized by mutations in genes that encode essential components of myelin and transcriptional regulators of Schwann cell development.

SOX10 is a Sry-related high mobility (HMG)-box transcriptional regulator that promotes differentiation of neural crest precursors into the glial lineage and is also involved in melanocyte differentiation [4]. The critical function of SOX10 in Schwann cell development and function is underscored by the occurrence of demyelinating neuropathies that result from SOX10 mutations [3]. SOX10 not only has a role in the commitment and early differentiation of neural crest cells into Schwann cell precursors, it is also required for their maturation into myelinating Schwann cells [4]. During early stages of differentiation, SOX10 promotes expression of low levels of myelin protein zero (MPZ), a major component of myelin that is specifically expressed in Schwann cells [5]. At later stages, SOX10 drives the myelination process through a stepwise feed forward mechanism. SOX10 first activates the POU homeo-domain transcription factor, OCT6 [6] which then cooperates with SOX10 to activate expression of the zinc finger transcriptional regulator, KROX20 [7]. In the next step, pro-myelinating Schwann cells transition to myelinating cells as SOX10 and KROX20 synergistically activate high levels of MPZ and the expression of genes encoding other components of myelin [8,9].

As a transcriptional activator, SOX10 and other SOX proteins bind to AT rich sequences in the minor groove and promote DNA bending [10]. The ability of SOX proteins to bend DNA and potentially change the architecture of target loci may promote transcription by facilitating interactions between target promoters and distal regulatory elements. However, the exact mechanisms by which SOX proteins promote transcription are poorly understood. A recent study suggests that SOX10 mediated transcriptional activation involves recruitment of SWI/SNF chromatin remodeling enzymes [11].

Mammalian SWI/SNF enzymes are evolutionarily conserved, multiprotein complexes that contain one of two ATPases, BRM or BRG1, and utilize the energy of ATP to disrupt chromatin structure and render chromatin permissive to the transcriptional machinery [12]. In vitro, chromatin remodeling is achieved by a core complex containing BRG1 or BRM, the INI1 subunit, BAF 170, and BAF 155, while *in vivo*, additional BRG1/BRM-associated factors (BAFs) are required for interactions with transcriptional activators and repressors which help recruit the SWI/SNF complex to specific genomic loci [13–19].

BRG1 and a number of other SWI/SNF components promote embryonic stem cell pluripotency and self renewal and are essential for mouse development [20–23]. SWI/SNF enzymes also function in cell cycle regulation, genome organization, and cellular differentiation [24]. Interactions between SWI/SNF components and lineage specific factors have been shown to drive muscle, neuron, adipocyte, melanocyte, myeloid, and more recently Schwann cell and oligodendrocyte differentiation [11,25–31]. During differentiation, SWI/SNF mediated chromatin remodeling promotes transcription of previously silent genes by facilitating stable pre-initiation complex formation and/or binding of gene specific activators or alternatively by promoting later stages of transcription [32–34]. Thus, the mechanisms by which SWI/SNF enzymes promote transcription depend on the context of the promoter.

Two recent studies indicate that conditional deletion of the BRG1 component of the SWI/SNF complex in mice results in loss of promyelinating transcription factors and severely inhibits Schwann cell differentiation [11,30]. In one study, BRG1 was found to activate SOX10 expression via interactions with NF-kappaB [30]. A different study found that BRG1 interacts with SOX10 to activate OCT6 and KROX20 expression [11]. However, neither study probed the direct requirement for SWI/SNF enzymes in the transcriptional regulation of myelin genes downstream of these transcriptional regulators.

In the current study, we tested the hypothesis that SWI/SNF enzymes are directly required to activate genes that encode components of peripheral myelin. In order to bypass the requirement for SWISNF enzymes in the activation of transcription factor expression, we utilized an in vitro model of differentiation in which SOX10, KROX20, or SOX10 together with KROX 20 were ectopically expressed in NIH 3T3-derived cells that inducibly express dominant negative versions of the BRM or BRG1 ATPase of the SWI/SNF complex [35]. Dominant negative BRM and BRG1 have mutations in the ATP binding site, thus are deficient for ATPase activity and cannot remodel chromatin. They have been shown to inhibit gene activation events that normally require SWI/SNF function [35]. We found that SOX10 can activate expression of two myelin genes, myelin protein zero (MPZ) and myelin basic protein (MBP) in these cells and that induction of dominant negative BRM or BRG1 inhibits expression of both myelin genes. We then focused on the requirement for the BRG1 ATPase because previous studies indicated that disruption of BRG1 in mice severely inhibits myelination while disruption of BRM has no obvious myelination defect [11,30,36]. In order to investigate the requirement for BRG1 in the transcriptional regulation of myelin genes, we ectopically expressed SOX10, KROX20, and SOX10 together with KROX20 in a cell line that inducibly expresses dominant negative BRG1. Expression of only KROX20 was not sufficient to activate expression of MPZ and MBP, but KROX20 together with SOX10 resulted in synergistic activation of these genes. Synergistic activation of MPZ and MBP was inhibited by dominant negative BRG1. We found that SOX10 promotes recruitment of BRG1 to the MPZ locus. SOX10 has previously been shown to activate an MBP reporter in NIH3T3 cells [37], however, to our knowledge, this is the first report that indicates SOX10 can reprogram NIH3T3 derived cells into cells that express endogenous myelin genes. We also demonstrate a direct requirement for SWI/SNF enzymes in the SOX10-mediated activation of myelin gene expression. In a complementary approach, we found that depletion of SOX10 in immortalized Schwann cells significantly decreased BRG1 occupancy at the MPZ locus. Furthermore, transient transfection of immortalized Schwann cells with dominant negative BRG1 decreased myelin gene expression. In combination, our data indicate that the chromatin remodeling domain of BRG1 is required to directly activate myelin gene expression in Schwann cells through SWI/SNF interactions with SOX10.

## Materials and Methods

### Cell Culture and expression Plasmids

Plasmids containing murine SOX10 cDNA [38] and KROX20 cDNA [39] were subcloned into pBabe retroviral vectors. Dominant negative BRM (H17) and dominant negative BRG1 (B22) cell lines inducibly express ATPase deficient, dominant negative alleles of BRM or BRG1 in a tetracycline dependent manner [35]. Cells were cultured in the presence (dominant negative expression OFF) or absence (dominant negative expression ON) of tetracycline for 3 days and were infected with pBabe-SOX10, pBabe-KROX20 retrovirus, or with retrovirus generated from the empty pBabe vector as previously described [25] for 30 hours. Cells were differentiated for 64 hours in a low serum medium containing 2% horse serum as described in [25]. Immortalized rat Schwann cells (S16) were purchased from ATCC and maintained in media with 10% fetal calf serum.

### RNA isolation and Quantitative Real Time PCR

Total RNA was isolated using Trizol (Invitrogen) and cDNA was prepared using the Qiagen Quantitect Reverse Transcription kit. Quantitative PCR (qPCR) was performed in SYBR Green master mix (Qiagen) with an Applied Biosystems 7500 PCR and analyzed with the SDS software as described [40]. Primers for mouse and rat MPZ were previously described in [41]: 5’-GCCCTGCTCTTCTCTTCTTT-3’ and 5’-CCAACACCACCCCATACCTA-3’, mouse MBP: 5’-TACCCTGGCTAAAGCAGAGC-3’ and 5’-GAGGTGGTGTTCGAGGTGTC-3’, mouse KROX20: 5’-TTGACCAGATGAACGGAGTG-3’ and 5’-ACCAGGGTACTGTGGGTCAA-3’. Primers to rat KROX20 were 5’-CCTGGGTGTGTGTACCATGT-3’ and 5’-GAGAGGAGGTGGAAGTGGTG-3’, rat MBP: 5’-GGCACGCTTTCCAAAATCT-3’ and 5-CGGGATTAAGAGAGGGTCTG-3’. In mouse cells, mRNA levels were normalized to mouse RPL7: 5’-GGAGGAAGCTCATCTATGAGAAGG-3’ and 5’-aagatctgtggaagaggaaggagc-3’. In rat cells, mRNA levels were normalized to rat 18S rRNA: 5’-AGTCCCTGCCCTTTGTACACA-3’ and 5’-GATCCGAGGGCCTCACTAAAC-3’.

### siRNA Knockdown

siRNA targeting rat SOX10 (5’-CUGCUGUUCCUUCUUGACCUUGC-3’) as reported in [42] and a non-targeting siRNA (5′-UUCUCCGAACGUGUCACGU-3′) were obtained from Dharmacon (Lafayette, CO) and transfected according to manufacturer’s instructions. Cells were harvested 96 hours post transfection.

### Cell extracts and immunoblot analysis

Western blots were performed as described [35]. Antisera to BRG1 was previously described [35]. The SOX10 (N-20) antibody was from Santa Cruz Biotechnology (Santa Cruz, CA, USA). The KROX20 antibody was from Covance (Princeton, New Jersey, USA). Antibodies to the FLAG epitope, total ERK1/2, and tubulin were from Cell Signaling Technology (Boston, MA, USA).

### Chromatin immunoprecipitations (ChIPs)

ChIPs were performed as described [40]. Primers that amplify the MPZ promoter and intron in mouse or rat were previously described [43]. ChIP signal at the MPZ locus was normalized to control IgG and to a control region with primers that amplify the mouse SCN2A1 promoter or rat Ig2a enhancer [43]

### Fluorescence-activated cell sorting (FACS)

Roughly 1x10^6^ cells were fixed with 100% ethanol for 1 hour, stained with PI-RNAse solution for 30mins and loaded on a FACS-Calibur (BD Biosciences, San Jose, CA, USA at the University of Toledo Flow Cytometry Core Facility). Data was analyzed using Cell Quest Pro (BD Biosciences).

### Statistical Analysis

Statistical significance was calculated by the Student’s t test when comparing two sets of data and a one way ANOVA followed by post-hoc testing when comparing more than two sets of data.

## Results

### Induction of myelin gene expression by SOX10 is inhibited by dominant negative BRM and BRG1

**Figure 1:**
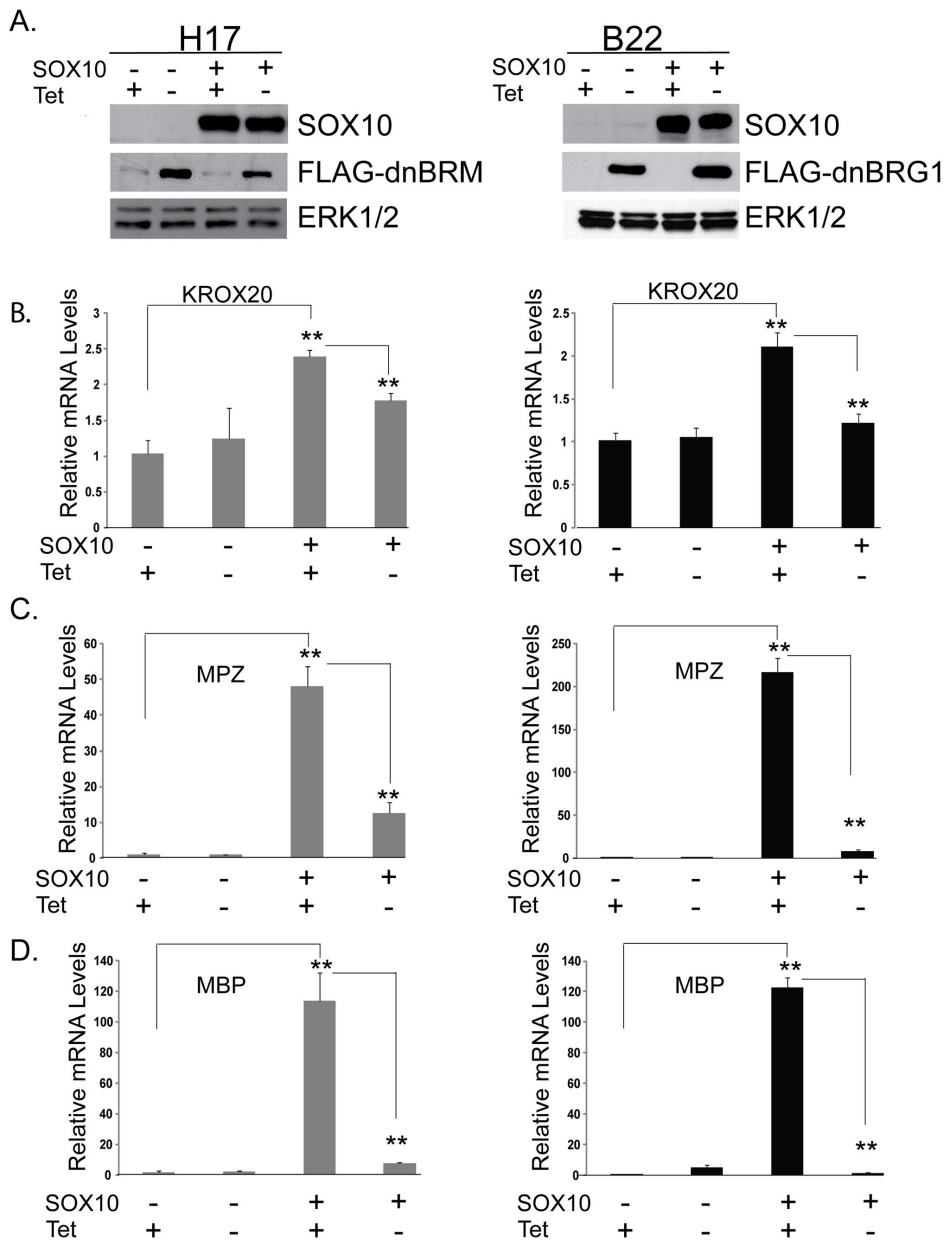
Dominant negative BRM and BRG1 inhibit SOX10-mediated activation of myelin genes. Cell lines that express dominant negative BRM or BRG1 were either infected with a pBabe control vector or pBabe-SOX10 in the presence or absence of tetracycline and then cultured in low serum media to promote differentiation. (A) Western Blot showing expression of SOX10 in cells that were cultured in the presence and absence of tetracycline and the expression of FLAG-tagged dominant negative BRM (left) in the H17 cell line and BRG1 (right) in the B22 cell line, when cells were cultured in the absence of tetracycline. Protein expression was detected from cell extracts and ERK1/2 was used as a loading control (B) Quantitative RT-PCR (qRT-PCR) of SOX10 target genes from pBabe or pBabe-SOX10 infected H17 (left) and B22 (right) cells. KROX20, MPZ, and MBP expression was normalized to expression of RPL7. The data are representative of at least four experiments and are the average of two independent experiments performed in triplicate. Standard error bars and statistical significance are shown (**p<0.01).

We previously described NIH3T3 derived cell lines, H17 and B22, that inducibly express dominant negative versions of BRM or BRG1 respectively under the control of the tetVP16 activator [35]. These cell lines have previously been used to test the requirement for SWI/SNF enzymes in tissue culture models of muscle, adipocyte, and melanocyte differentiation promoted by ectopic expression of the appropriate lineage specific factors [25,28,32]. In order to determine whether SOX10 can convert these cells into myelin gene expressing cells and to test the requirement for SWI/SNF enzymes in the activation of these genes, we introduced SOX10 by retroviral infection into a dominant negative BRM cell line (H17), and a dominant negative BRG1 cell line (B22) that had been grown in the presence or absence of tetracycline and then cultured in low serum media to promote differentiation. Figure 1A shows ectopic expression of the SOX10 protein and FLAG-tagged dominant negative BRM and BRG1 expression in H17 and B22 cells respectively, when the cells were cultured in the presence or absence of tetracycline.

Interestingly, we found that SOX10 modestly but significantly increased expression of KROX20 at the mRNA level by approximately two fold in H17 (left) and B22 (right) cells that were differentiated in the presence of tetracycline (dominant negative BRM and BRG1 off (Figure 1B). Activation of KROX20 was partially inhibited by dominant negative BRM and BRG1 when cells were differentiated in the absence of tetracycline (Figure 1B).

SOX10 robustly induced the expression of two myelin genes, myelin protein zero (MPZ) (Figure 1C) and myelin basic protein (MBP) (Figure 1D) in H17 and B22 cells that were differentiated in the presence of tetracycline (dominant negative BRM and BRG1 off). However, expression of these genes was dramatically inhibited by the presence of dominant negative BRM and BRG1 when cells were differentiated in the absence of tetracycline. These results indicate that SOX10 can reprogram NIH 3T3 cells to express myelin genes by a mechanism that is dependent on SWI/SNF enzymes.

### Synergistic activation of myelin genes by SOX10 and KROX20 requires BRG1

**Figure 2:**
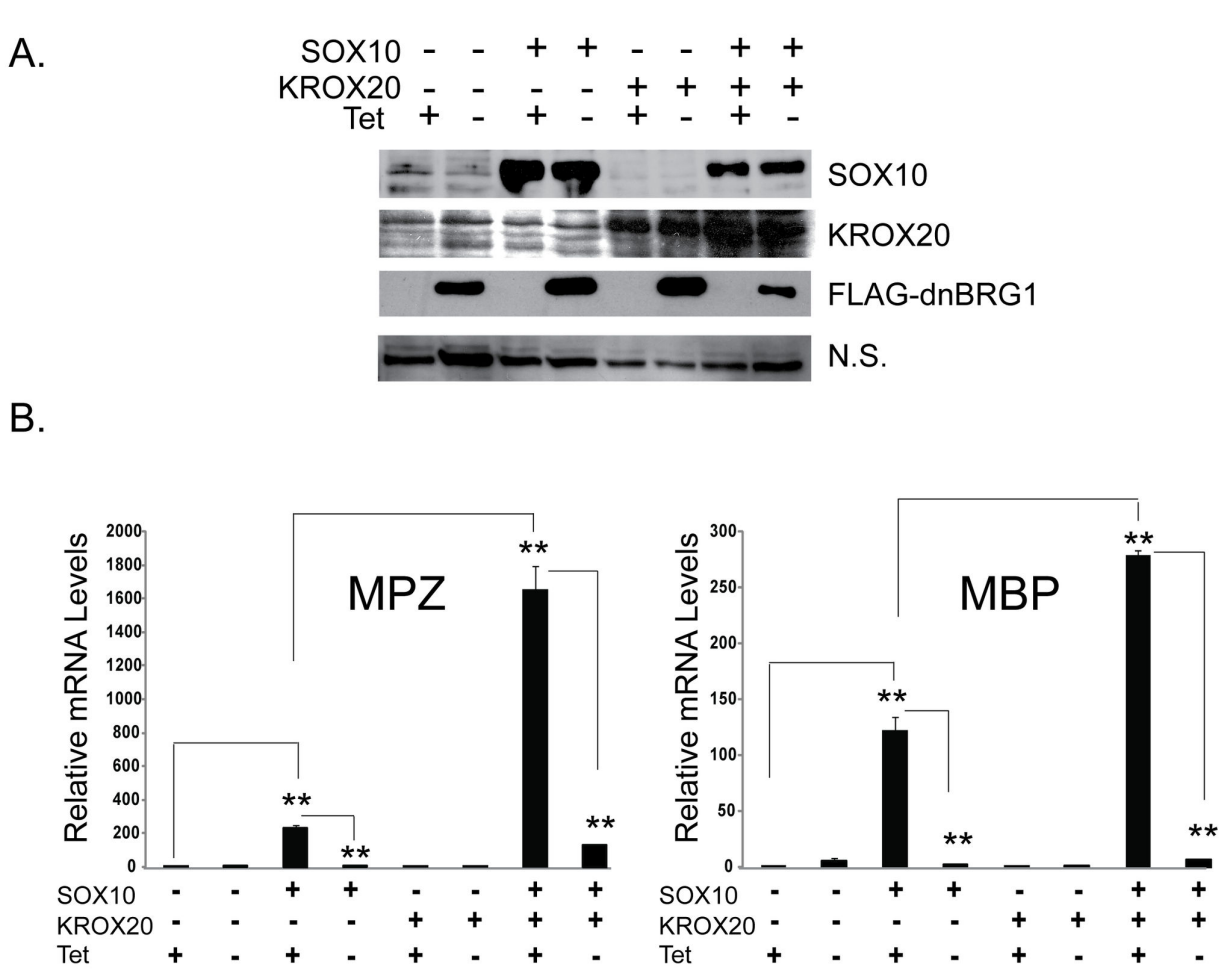
Dominant negative BRG1 inhibits synergistic activation of myelin genes by SOX10 and KROX20. Cell lines that express dominant negative BRG1 were infected with an empty vector, pBabe-SOX10, pBabe-KROX20, or pBabe-SOX10 together with pBabe-KROX20 in the presence or absence of tetracycline and then cultured in low serum media to promote differentiation. (A) Western Blot showing expression of SOX10 and KROX20 in the presence and absence of tetracycline and the expression of FLAG-tagged dominant negative BRG1 in the B22 cell line when cells were cultured in the absence of tetracycline. Protein expression was detected from nuclear extracts and a non-specific band was used as a loading control. (B) Quantitative RT-PCR (qRT-PCR) of SOX10 target genes from pBabe or pBabe-SOX10, pBabe-SOX10, pBabe-KROX20, or pBabe-SOX10 together with pBabe-Krox20 infected cells. Expression of MPZ and MBP was normalized to expression of RPL7. The data are representative of greater than three experiments and are the average of two independent experiments performed in triplicate. Standard error bars and statistical significance are shown (**p<0.01).

Neural crest precursors, and pro-myelinating Schwann cells express low levels of some myelin genes and the expression of these genes is highly up-regulated during myelination [44]. Myelination involves a transcriptional cascade by which SOX10 first activates KROX20 expression and then cooperates with KROX20 to synergistically activate the promoters of a subset of myelin genes. We focused on the BRG1 ATPase of the SWI/SNF complex, because BRG1 has been found to be required for the formation of myelin by Schwann cells in mice [11,30]. To determine if the observed requirement for BRG1 in the activation of MPZ and MBP expression (Figure 1 C and D) is solely due to the requirement for BRG1 in the activation of KROX20 (Figure 1B) or due to a requirement for BRG1 in the direct activation of these genes, we expressed SOX10 alone, KROX20 alone, and SOX10 together with KROX20 in B22 cells that were differentiated in the presence or absence of tetracycline (Figure 2A). Our data indicate that expression of KROX20 alone is not sufficient to activate MPZ and MBP expression in NIH3T3 derived cells (Figure 2B). Interestingly, when cells were differentiated in the presence of tetracycline (dominant negative BRG1 off), MPZ mRNA levels were approximately eight-fold higher and MBP mRNA levels were approximately three-fold higher in cells expressing both KROX20 and SOX10 compared to cells expressing only SOX10. Expression of dominant negative BRG1 caused a significant inhibition of the synergistic activation of MPZ and MBP expression by SOX10 and KROX20. Thus, the inhibitory effect of dominant negative BRG1 on MPZ and MBP expression was not rescued by KROX20 expression. Furthermore, these data suggest that SWI/SNF enzymes are not only required downstream of KROX20 for activation of low levels of MPZ and MBP expression by SOX10 but are also required for the synergistic activation of MPZ and MBP expression by SOX10 and KROX20.

### Cell cycle arrest occurs independently of SWI/SNF enzymes

**Figure 3:**
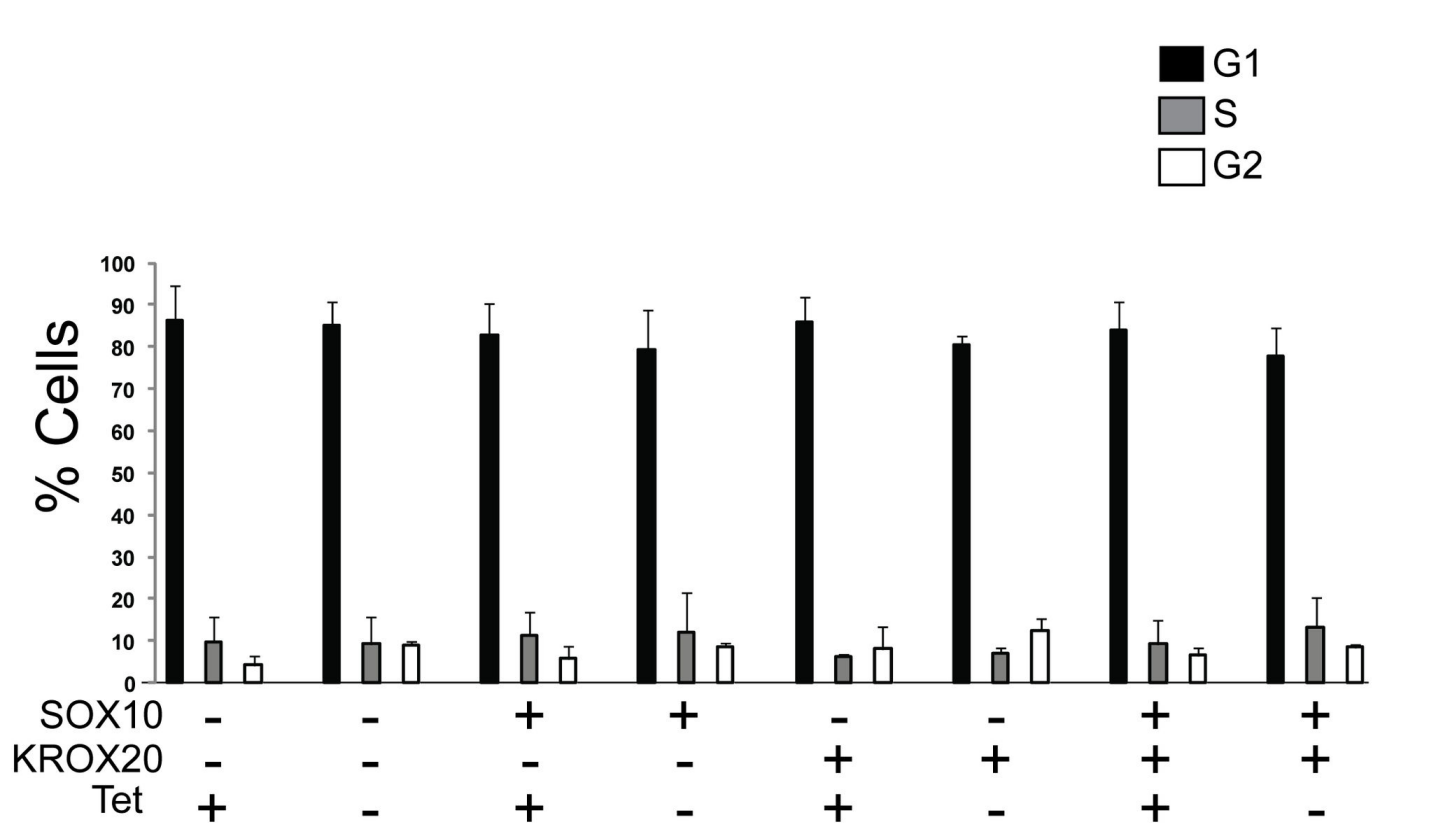
FACS analysis of differentiated B22 cells. B22 cells were infected with an empty vector, pBabe-SOX10, pBabe-KROX20, or pBabe-SOX10 together with pBabe-KROX20 in the presence or absence of tetracycline and differentiated in low serum media. Propidium iodine stained samples were FACS sorted and analyzed using Cell Quest Pro (BD Biosciences). The data are the average of three independent experiments. Standard error bars are shown.

Cessation of DNA synthesis and withdrawal from the cell cycle is coordinated with myelination during Schwann cell differentiation [45]. In order to determine whether SWI/SNF enzymes are required for cell cycle withdrawal in this tissue culture model of differentiation, we cultured cells expressing either SOX10, KROX20, or SOX10 with KROX20 in low serum media in the presence or absence of dominant negative BRG1. At the end of differentiation, cells were stained with propidium iodide to determine the number of cells in the different phases of the cell cycle. Figure 3 indicates that expression of dominant negative BRG1 did not affect the ability of SOX10 and KROX20 expressing cells to arrest in the G1 phase of the cell cycle. This suggests that the catalytic domain of BRG1 is required for activation of myelin gene expression by these transcriptional regulators but is not required to promote cell cycle withdrawal under these conditions.

Ectopic expression of SOX10 promotes recruitment of SWI/SNF enzymes to the MPZ regulatory region in NIH 3T3 cells

**Figure 4:**
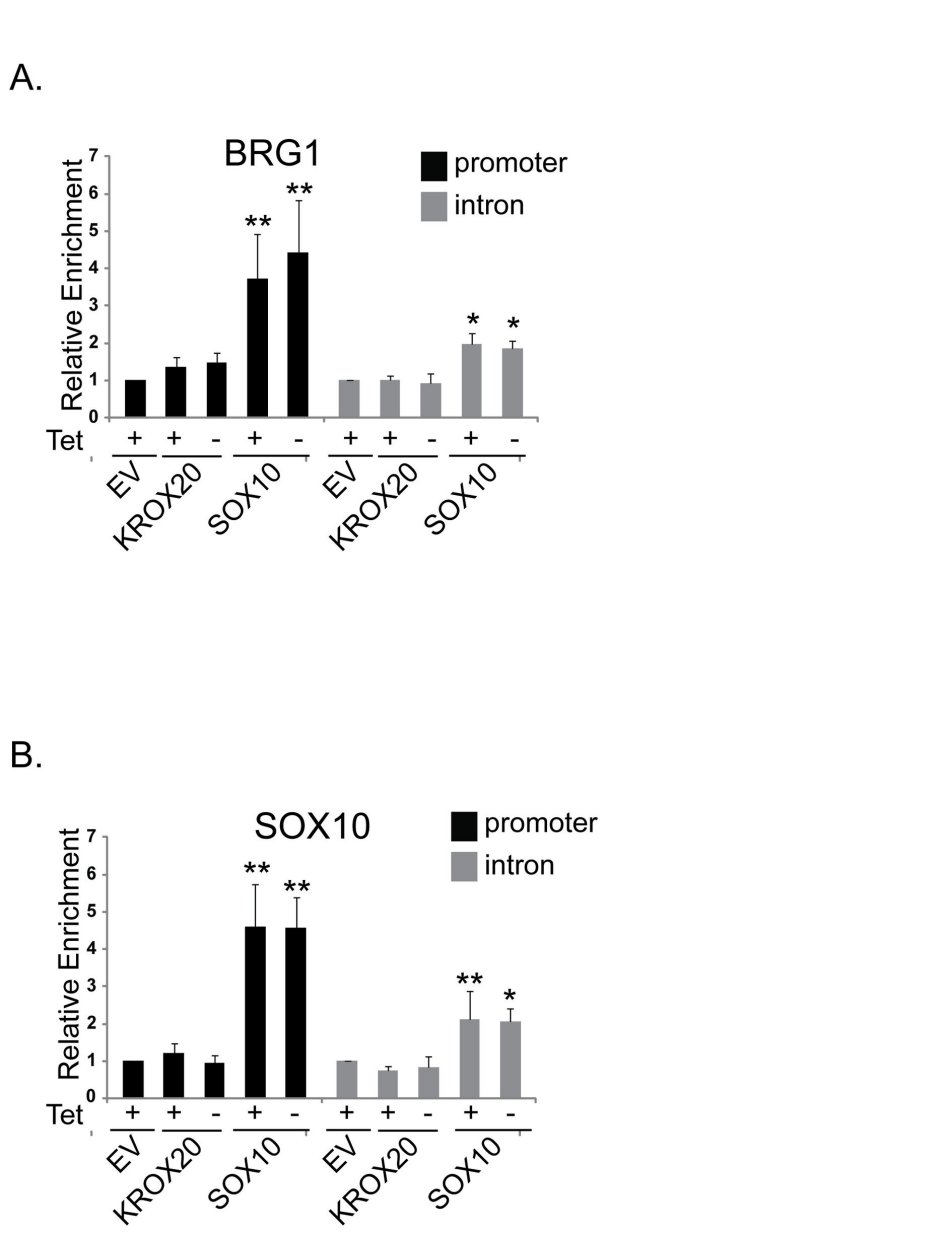
BRG1 is recruited to the MPZ promoter as a result of SOX10 mediated differentiation. Chromatin immunoprecipitations (ChIPs) were performed with control IgG, antisera to BRG1, or an antibody to SOX10 on chromatin from B22 cells that were infected with empty vector (EV), KROX20, or SOX10 retrovirus and cultured as described in Figure 1. Enrichment at the MPZ promoter and intron is relative to control IgG and normalized to a control region, SCN2A1. There was minimal variation in the ChIP signal at the SCN2A1 region. (A) Detection of BRG1 interactions with the MPZ promoter and intron. (B) Detection of SOX10 interactions with the MPZ promoter and intron. This data are representative of at least three experiments and are the average of two independent experiments that were assayed four or more times, each in triplicate. Standard errors bars and statistical significance are shown (**p<0.01, * p<0.05).

MPZ is a Schwann cell specific component of myelin that is expressed at basal levels early in neural crest development and is highly activated during Schwann cell myelination [44]. A 1.1 kb region of the MPZ promoter that contains high affinity SOX10 binding sites is sufficient for basal expression of MPZ while additional sequences including a region in the first intron that contains low affinity SOX10 binding sites as well as a KROX20 binding site are important for high levels of MPZ expression [8,41]. In order to elucidate the mechanisms by which components of the SWI/SNF complex are recruited to the MPZ promoter, we performed chromatin immunoprecipitations (ChIPs) in control cells expressing empty vector (EV), and in cells expressing KROX20 or SOX10. We found that ectopic expression of KROX20 alone did not significantly increase BRG1 association with either the promoter or intronic region of MPZ when compared to the empty vector control (Figure 4A). In contrast, ectopic expression of SOX10 resulted in a significant increase in the recruitment of BRG1 to the MPZ promoter and to a lesser but significant extent to the MPZ intron (Figure 4A). BRG1 enrichment on these sites was not significantly affected by the expression of dominant negative BRG1 in cells that were cultured without tetracycline. This is consistent with previous findings that dominant negative BRG1 associates with other components of the SWI/SNF complex and is recruited to its target loci, but is not functional at these loci [28,33].

SOX10 enrichment on the MPZ promoter was substantially higher than on the MPZ intron in B22 cells that ectopically express SOX10 (Figure 4B). Thus, ectopic expression of SOX10 in NIH3T3 derived cells results in SOX10 association with the same regions of the MPZ locus as was previously observed in myelinating rat sciatic nerve [43]. Furthermore, we found that SOX10 occupancy at these sites was not significantly affected by the expression of dominant negative BRG1. In combination, these data suggest that the mechanism by which SOX10 reprograms NIH3T3 cells to express this myelin gene involves binding to the MPZ regulatory regions and recruiting BRG1.

### SOX10 promotes BRG1 recruitment to MPZ regulatory regions in immortalized Schwann cells

**Figure 5:**
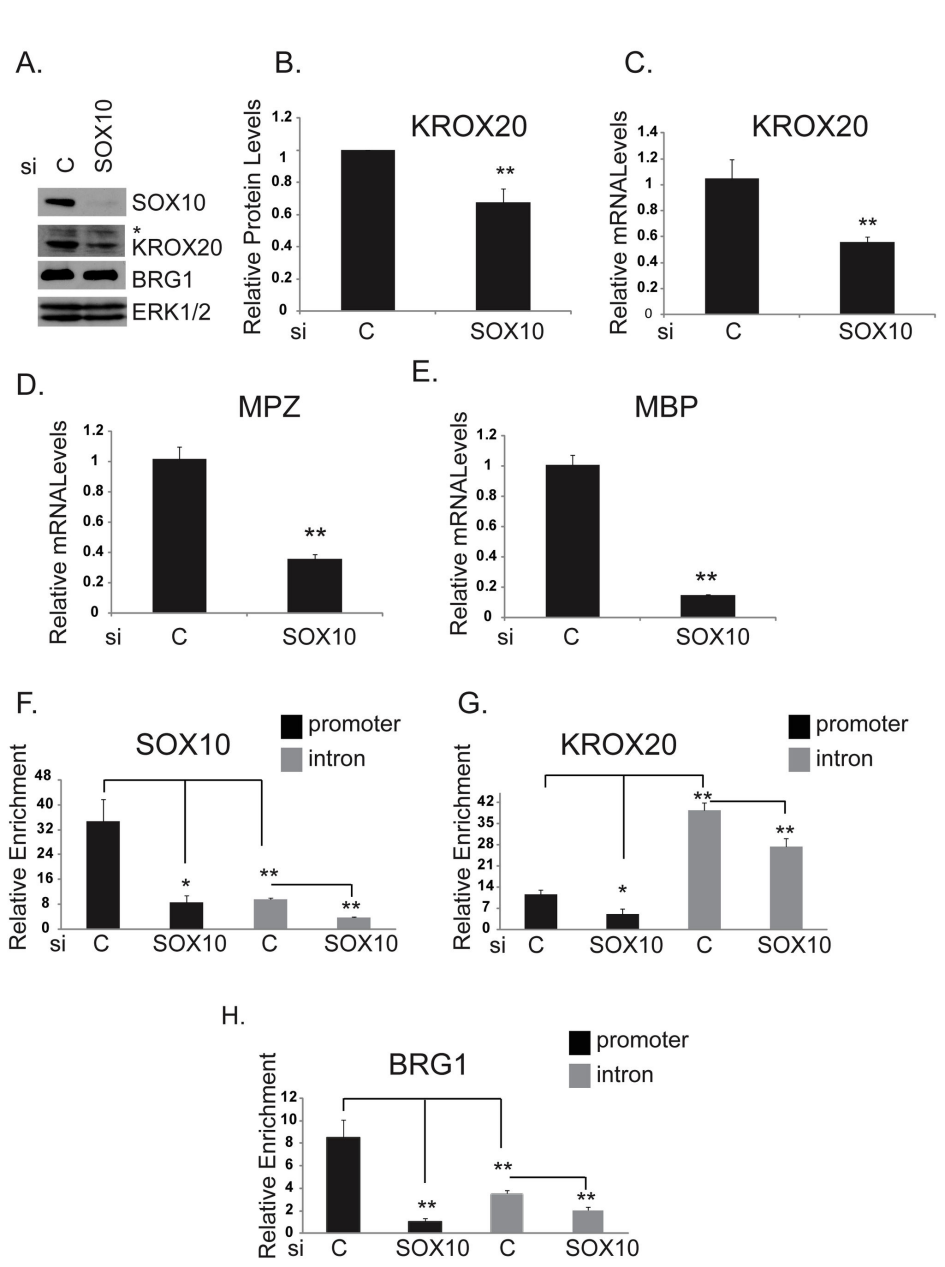
SOX10 is required to recruit BRG1 to the MPZ promoter in S16 Schwann cells. A. Cell extracts were prepared from S16 cells that were transfected with siControl or siSOX10 and subjected to Western analysis with antibodies to SOX10, KROX20, and antisera to BRG1 (The starred top band (*) in the KROX20 Western may be a non-specific band. It was not used in the quantitation of KROX20 expression shown in Figure 5B. ERK1/2 is shown as a loading control. B. Quantitation of KROX20 protein levels relative to ERK1/2 protein levels by ImageJ software. The data are the average of three independent experiments. Standard error bars and statistical significance are shown (**p<0.01). C–E. Quantitative RT-PCR (qRT-PCR) of KROX20, MPZ, and MBP mRNA levels. F–H. ChIPs were performed with control IgG, antibodies to SOX10 or KROX20, or antisera to BRG1 on chromatin from S16 cells that were transfected with siControl or siSOX10. Enrichment on the MPZ promoter and intron is relative to control IgG and normalized to a control region, Ig2a enhancer. There was minimal variation in the ChIP signal at the Ig2A enhancer. (F) Detection of SOX10 interactions with the MPZ promoter. (G) Detection of KROX20 interactions with the MPZ promoter. (H) Detection of BRG1 interactions with the MPZ promoter. The data are representative of three independent experiments that were assayed three times. Standard errors bars and statistical significance are shown (**p<0.01, *p<0.05).

A recent report demonstrated a requirement for BRG1 in Schwann cell differentiation by virtue of SOX10 dependent recruitment of BRG1 to the regulatory regions of genes encoding the transcription factors, OCT6 and KROX20, in myelinating sciatic nerve and in immortalized Schwann cells [11]. However, this report did not investigate whether SOX10 recruits BRG1 to the regulatory regions of genes that encode components of myelin, such as MPZ. We performed ChIPs to assess if SOX10 also recruits BRG1 to the MPZ locus in immortalized Schwann cells (S16) that were transfected with control siRNA or with siSOX10. Western blotting indicated that siSOX10 efficiently depleted SOX10. Depletion of SOX10 abrogated KROX20 expression at the protein level (Figure 5A and B) but did not affect the expression of BRG1 (Figure 5A). SOX10 depletion also abrogated KROX20 mRNA levels (Figure 5C) and to a greater extent, MPZ and MBP mRNA levels (Figure 5D and E).

Consistent with a previous study in myelinating sciatic nerve [43] as well as our data in B22 cells (Figure 4B), we detected strong enrichment of SOX10 on elements in the MPZ promoter and weaker enrichment on elements in the first intron of MPZ in these cells (Figure 5F). Transfection with siSOX10 significantly reduced SOX10 enrichment on both these regulatory regions. In contrast to SOX10, KROX20 was strongly associated with the intron and only weakly associated with the promoter (Figure 5G). Mutation of SOX10 binding sites in the MPZ intron was shown not to interfere with KROX20 binding on naked DNA templates [46], however it is not known whether SOX10 is required for KROX20 to bind its recognition sites at the endogenous MPZ locus. We found that depletion of SOX10 resulted in a small but significant decrease in KROX 20 association with both the MPZ promoter and intron (Figure 5G). However, due to the effects of SOX10 depletion on KROX20 protein levels (Figure 5A and 5B), it is unclear whether SOX10 is directly required for KROX20 to associate with these regions or the effects are a reflection of reduced KROX20 levels. Interestingly, as we observed in B22 cells (Figure 4A), BRG1 was highly enriched on the MPZ promoter in control cells and to a lesser extent on the intron (Figure 5H). Occupancy of BRG1 was dependent on SOX10, as demonstrated by the significant decrease in enrichment of BRG1 on both the promoter and intron in cells that were depleted of SOX10. Thus, the enrichment profile of BRG1 closely parallels that of SOX10 on these MPZ regulatory regions and is dependent on SOX10 expression.

### Dominant negative BRG1 abrogates myelin gene expression in immortalized Schwann cells

**Figure 6:**
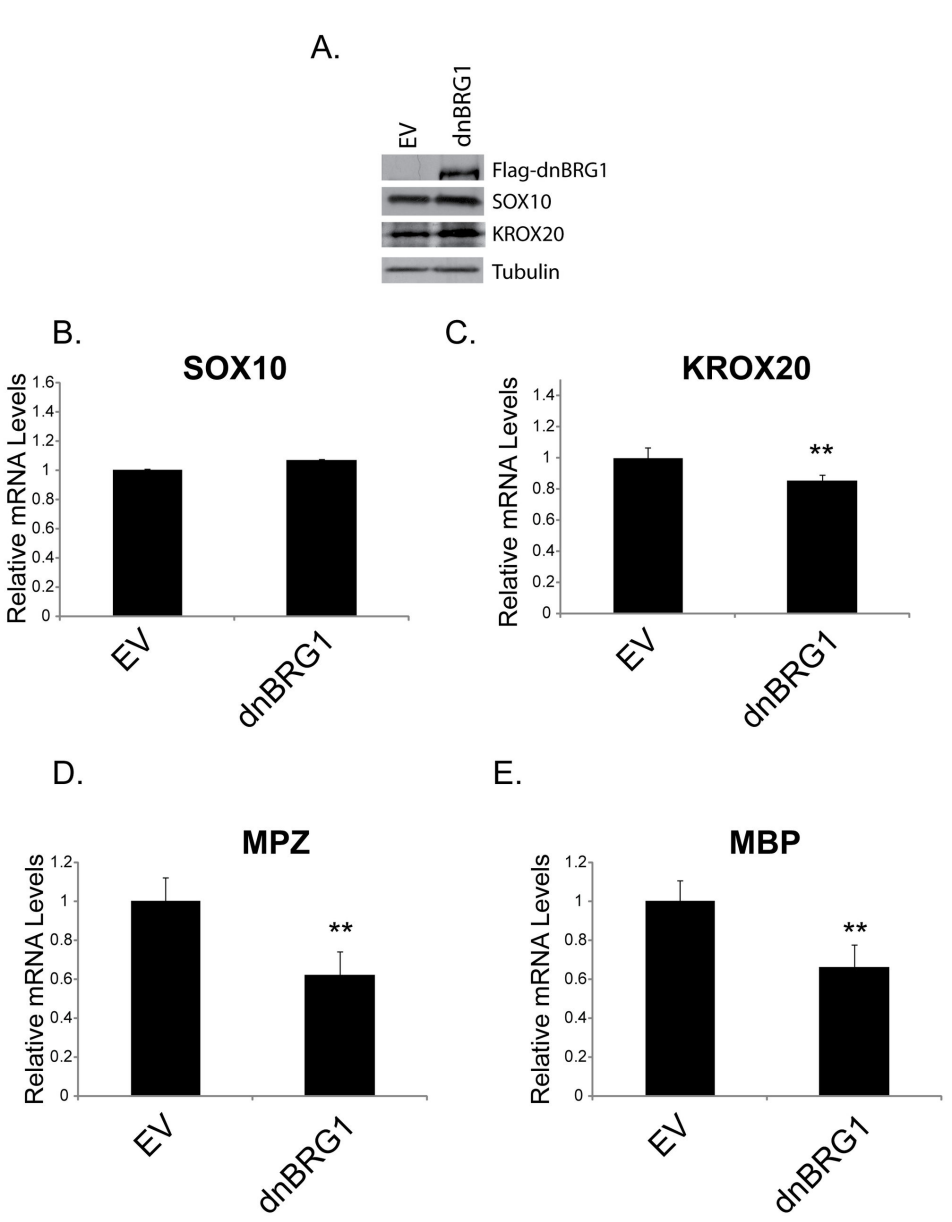
Dominant negative BRG1 inhibits expression of SOX10 target genes in S16 Schwann cells. S16 cells were transfected with GFP (not shown), empty vector (EV), or pBabe-dominant negative BRG1 (dnBRG1). Transfection efficiency was monitored by GFP and was determined to be approximately 30%. (A) Cell extracts were prepared and subjected to Western analysis with antibodies to the Flag epitope, SOX10, or KROX20. Tubulin is shown as a loading control. Quantitative RT-PCR (qRT-PCR) of SOX10 (B), KROX20 (C), MPZ (D), and MBP (E) from empty vector (EV) or pBabe-dnBRG1 transfected cells. Expression of each gene was normalized to that of 18S rRNA. The data are the average of three independent experiments performed in triplicate. Standard error bars are shown (**p<0.01).

Mice with a Schwann cell specific deletion of BRG1 display a dramatic reduction in the number of myelinating KROX20 positive cells and a decrease in the expression of myelin genes [11]. However, it was not determined from this study if the catalytic domain of BRG1 is required for expression of these genes. In order to determine if the catalytic domain of BRG1 is required for maintenance of Schwann cell specific gene expression, we ectopically expressed a dominant negative version of BRG1 into the S16 immortalized Schwann cell line. We found that transient transfection of S16 cells with dominant negative BRG1 did not decrease SOX10 or KROX20 at the protein level (Figure 6A). Dominant negative BRG1 also did not significantly inhibit SOX10 expression at the mRNA level (Figure 6B) and had a small but significant inhibitory effect on KROX20 mRNA levels (Figure 6C). Expression of MPZ, and MBP was significantly inhibited by dominant negative BRG1 (Figure 6D, E). Thus, the chromatin remodeling activity of BRG1 is likely to be required for maintenance of myelin gene expression in differentiated Schwann cells.

## Discussion

It is well established that cellular differentiation is highly dependent on SWI/SNF chromatin remodeling activity to render previously silent lineage specific loci permissive for transcription [24]. Multiple studies have found that an important mechanism by which SWI/SNF enzymes regulate lineage specific gene expression is through interactions with transcriptional regulators that likely recruit SWI/SNF enzymes to the relevant genomic sites [26,28,32,47]. Studies on muscle differentiation suggest that SWI/SNF recruitment is precisely orchestrated to establish the temporal patterning of gene expression that ultimately transforms undifferentiated cells into terminally differentiated cells [33,48]. By somewhat different mechanisms, SWI/SNF activity is also modulated during neurogenesis to regulate the timing of terminal differentiation [49]. Thus, there are likely to be lineage specific mechanisms that operate through chromatin remodeling to precisely regulate the timing of cellular differentiation. However, very little is known about these mechanisms in other lineages.

**Figure 7:**
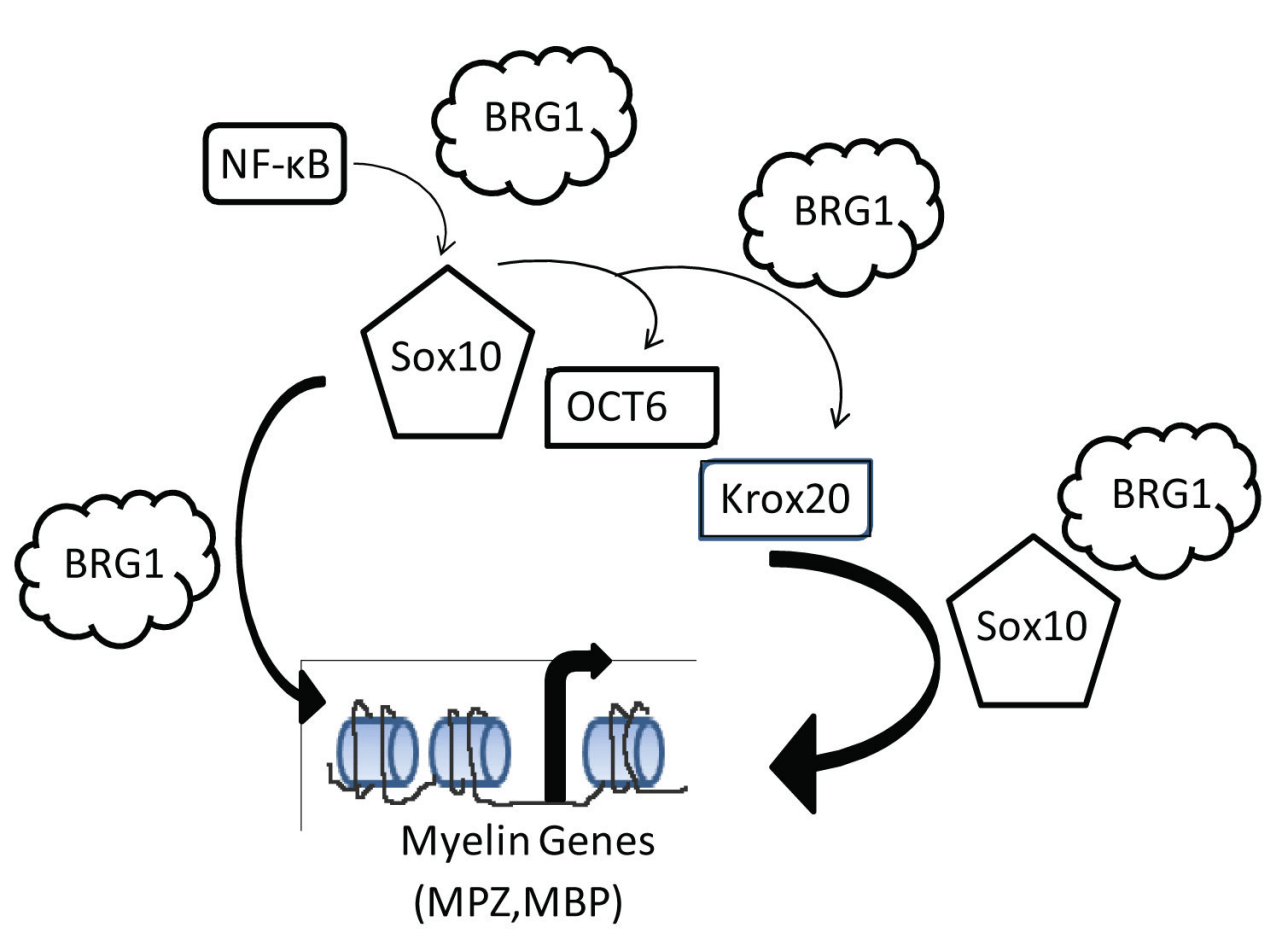
The requirement for BRG1 during Schwann cell differentiation and myelination. A model illustrating the stepwise requirement for BRG1 during Schwann cell differentiation and myelination based on the current study as well as two recent studies [11,30]..

Early studies implicated SWI/SNF enzymes in neural crest differentiation and gliogenesis [50,51]. The requirement for SWI/SNF enzymes in the specification of neural crest precursors into oligodendrocytes of the central nervous system and Schwann cells of the peripheral nervous system as well as the transcriptional interactions that regulate SWI/SNF recruitment have only recently been elucidated [11,30,31]. During Schwann cell differentiation, the BRG1 component of the SWI/SNF complex was found to interact with NFkappaB to activate SOX10 expression and in turn to interact with SOX10 to activate the expression of the transcriptional regulators, OCT6 and KROX20 [11,30]. Our study uncovers a requirement for BRG1 in the direct regulation of genes encoding components of myelin through BRG1 interactions with SOX10. Thus, SWI/SNF enzymes are extensively required for the activation of multiple classes of genes that ultimately result in myelin gene expression in terminally differentiated Schwann cells (Figure 7).

Master regulators of differentiation have been identified by their ability to convert heterologous cells into cells that express lineage specific genes. We found that SOX10 can activate two myelin genes, one of which is a Schwann cell specific marker. Interestingly dominant negative BRM and BRG1 severely inhibited activation of these genes. We focused on BRG1 for the remainder of our studies because mice with conditional disruption of BRG1 exhibit myelination defects while BRM disrupted mice do not [11,30,36]. Although our data indicate that BRM may also promote myelin gene expression, the requirement for BRM is likely to be at least partially compensated by BRG1 *in vivo*.

Our study in transdifferentiated B22 cells indicates that SOX10 promotes recruitment of BRG1 to two regulatory regions of the MPZ locus. Importantly, we found that KROX20 in the absence of SOX10 cannot activate myelin gene expression, nor can KROX20 promote the recruitment of BRG1 to these two MPZ regulatory sites. This is consistent with a previous study in which KROX20-mediated activation of MPZ transcription depended on SOX10 binding sites [43]. Although KROX20 cannot promote BRG1 recruitment in the absence of SOX10, our data do not rule out that KROX20 either directly or indirectly contributes to BRG1 recruitment in conjunction with SOX10. Alternatively, SWI/SNF mediated chromatin remodeling may be required for KROX20 to bind to cognate sites in the MPZ regulatory region if they are embedded in repressive chromatin structure. Ongoing studies are currently dedicated toward elucidating the dependency of KROX20 binding on BRG1 and the dependency of BRG1 recruitment on KROX20 and on characterizing the chromatin structure of the MPZ regulatory region.

BRG1 recruitment to the MPZ promoter and intron was also dependent on SOX10 in immortalized Schwann cells. Interestingly, although the enrichment profile of SOX10 and BRG1 is similar in B22 cells that express SOX10 and in immortalized Schwann cells that express SOX10, there appears to be greater enrichment of these factors on the MPZ promoter in Schwann cells (compare Figure 4A and 4B and Figure 5F and 4H). Dominant negative BRG1 dramatically inhibited SOX10 mediated activation of MPZ as well as synergistic activation of MPZ by SOX10 and KROX20 in B22 cells (Figure 1C) but had a modest effect on MPZ expression in Schwann cells (Figure 6D). The less dramatic effect of dominant negative BRG1 on MPZ expression in Schwann cells may reflect the transient nature of dominant negative BRG1 expression and possibly a need to compete with higher levels of wildtype BRG1 on the MPZ promoter. However, it is important to note that *induction* of MPZ expression was assayed in B22 cells, whereas *maintenance* of MPZ expression was assayed in Schwann cells. Studies on the PHO and GAL genes in yeast suggest that SWI/SNF enzymes are required to increase the initial rates of transcription induction [52,53]. Thus, although BRG1 contributes to the maintenance of myelin gene expression, there may be a greater requirement for SWI/SNF mediated chromatin remodeling in promoting the initial rate of MPZ activation. In combination, these data demonstrate a direct role for BRG1 in the regulation of myelin gene expression that is dependent on SOX10.

SOX10 may be classified as a master regulator or lineage determination factor of Schwann cell differentiation in part through its ability to promote the recruitment of SWI/SNF enzymes and thereby activate myelin gene expression in heterologous cells. However, since SOX10 also promotes differentiation of neural crest precursors into other lineages, including oligodendrocyte and melanocyte, the role of SOX10 as a master regulator or determination factor of Schwann differentiation is likely to be modulated by additional levels of regulation.

The transition from an immature promyelinating cell to a myelinating cell is regulated by the concerted activity of SOX10 and several other transcription factors. During myelination, SOX10 activates expression of the transcriptional regulator, KROX20 and then synergizes with KROX20 to activate genes encoding myelin components [54]. We found that although KROX20 alone cannot activate MPZ or MBP expression in NIH3T3 derived cells, ectopic expression of KROX20 together with SOX10 leads to synergistic activation of MPZ and MBP in NIH3T3 derived cells, thus mimicking the myelination step during Schwann cell differentiation. Furthermore, synergistic activation of MPZ and MBP by SOX10 and KROX20 was inhibited by dominant negative BRG1, suggesting that SWI/SNF complexes are directly required for promoting basal expression of MPZ and MBP as occurs in neural crest precursors and pro-myelinating Schwann cells and for activated levels of transcription as occurs in myelinating Schwann cells [44]. Thus, BRG1 regulates myelin gene expression at multiple phases of Schwann cell differentiation.

SOX10 is utilized at multiple steps during neural crest differentiation and is required for differentiation into pro-myelinating as well as myelinating cells, in addition to neural crest differentiation into oligodendrocyte and melanocyte lineages [55]. The requirement for this lineage determination factor for late stages of Schwann cell differentiation is in contrast to the requirement for the lineage determination, factor, MYOD during late stages of muscle differentiation. MYOD is a master regulator of muscle differentiation that can activate the muscle specific gene expression program in heterologous cells [56,57]. MYOD commits precursors to the muscle lineage and is critical for early stages of muscle differentiation, including the activation of two transcriptional regulators, myogenin and MEF2 [58]. This cascade of transcription factor activity is then critical for controlling the timing of late muscle specific gene expression in part by regulating BRG1 recruitment to the regulatory regions of late muscle specific genes [48,59]. Elucidation of the role of other transcriptional regulators for the recruitment of SWI/SNF components to Schwann cell specific loci is likely to provide insight into the mechanisms that regulate temporal patterning of gene expression during early neural crest differentiation into several different lineages as well as for myelination of Schwann cells.

By utilizing a dominant negative approach in both a reprogramming model of differentiation as well as in immortalized Schwann cells, our work strongly suggests that SWI/SNF chromatin remodeling activity is required for SOX10 mediated activation of genes that encode myelin constituents. Future studies will investigate the types of chromatin modifications that SWI/SNF enzymes catalyze for the activation of these genes and how they impinge upon the DNA bending properties of this multifunctional HMG domain transcriptional regulator.
